# Metabolomic Signatures of Relapse and Survival in AML Patients Receiving Allogeneic Hematopoietic Stem Cell Transplantation

**DOI:** 10.3390/hematolrep18020027

**Published:** 2026-04-07

**Authors:** Igor Novitzky-Basso, Changjiang Xu, Caden Chiarello, Julie A. Reisz, Angelo D’Alessandro, Gary D. Bader, Jonas Mattsson, Courtney Jones

**Affiliations:** 1 Division of Medical Oncology and Hematology, Princess Margaret Cancer Centre, Toronto, ON M5G 2M9, Canada; 2 Department of Medicine, University of Toronto, Toronto, ON M5S 1A1, Canada; 3 The Donnelly Centre, University of Toronto, Toronto, ON M5S 1A1, Canada; 4 Department of Biochemistry and Molecular Genetics, University of Colorado Anschutz Medical Campus, Aurora, CO 80045, USA; 5 Division of Experimental Hematology and Cancer Biology, Cincinnati Children’s Hospital Medical Center, Cincinnati, OH 45229, USA

**Keywords:** metabolomics, allogeneic stem cell transplant, acute myeloid leukemia

## Abstract

**Objectives**: Allogeneic stem cell transplantation (HSCT) is curative in acute myeloid leukemia (AML) but is limited by relapse and non-relapse mortality (NRM). Metabolomic prognostic value is unclear. We assessed whether plasma metabolite profiles at diagnosis, pre-transplant, and post-transplant are associated with overall survival (OS) and cause-specific mortality. **Methods**: We retrospectively analyzed plasma metabolites from 63 AML patients undergoing HSCT (263 samples). **Results**: Higher levels of valine (hazard ratio [HR] 24.454), citrulline (HR 20.478), 5-oxoproline (HR 11.766), and glutamine (HR 8.701) associated with higher NRM, while inosine diphosphate (HR 0.091) and pyridoxamine-5′-phosphate (HR 0.313) associated with lower NRM. For relapse-related mortality (RRM), higher levels of phenylalanine (HR 26.585), leucine/isoleucine (HR 10.755), indolepyruvate (HR 7.676), and creatinine (HR 13.874) were associated with higher RRM, while trans-4-hydroxy-L-proline (HR 0.101) was associated with lower RRM. Higher post-transplant ornithine (HR 0.063), 3-sulfocatechol (HR 0.590), and indole-3-acetate (HR 0.359) were associated with improved OS. Mixed-effects modelling identified lower dehydroascorbate and citrate in relapsed patients, with dehydroascorbate remaining significant after false discovery rate adjustment. **Conclusions**: Metabolomic profiling nominated candidate metabolites for validation in larger prospective studies and elucidated mechanistic pathways, potentially informing novel interventions or risk-adapted monitoring strategies in HSCT.

## 1. Introduction

Allogeneic hematopoietic stem cell transplantation (HSCT) is a potentially curative therapy for a variety of haematologic malignancies and selected non-malignant disorders [1]. Over the past decades, advances in donor selection, conditioning regimens, and supportive care have improved transplant success. However, outcomes remain limited by significant complications, notably graft-versus-host disease (GvHD), relapse of the underlying disease, and transplant-related (non-relapse) mortality (NRM) [2]. Acute GvHD, resulting from donor T cells attacking host tissues, affects ~30–50% of recipients and can cause severe morbidity or death despite immunosuppressive therapy [3]. Chronic GvHD, as well as opportunistic infections and organ toxicities, also contribute significantly to late NRM. Relapse of the original malignancy remains the single most frequent cause of treatment failure after HSCT. Clinically, these endpoints frequently interact and trade off against one another (for example, intensified immunosuppression to control GvHD may compromise anti-leukemic immune effects, graft-versus-leukemia (GvL), and increase infectious vulnerability), underscoring the need for earlier and more granular risk assessment. Because overall survival after HSCT is a composite of relapse-related mortality and NRM, biomarker associations with overall survival may reflect differing underlying event types; complementary cause-specific analyses can help distinguish whether a candidate marker primarily tracks relapse biology, treatment toxicity, or inflammatory complications, rather than overall prognosis alone.

In recent years, the landscape of allogeneic HSCT has been transformed by the widespread adoption of post-transplant cyclophosphamide (PTCy) as the standard of care for graft-versus-host disease (GvHD) prophylaxis. Mechanistically, PTCy effectively uncouples GvHD from the graft-versus-leukemia (GvL) effect by selectively depleting proliferating alloreactive T cells while sparing non-dividing regulatory T cells (Tregs) and memory T cells, which are essential for immune tolerance and viral defence [4,5]. While the clinical efficacy of PTCy is well-established, the specific systemic metabolic environment required to support this unique pattern of immune reconstitution remains unknown. Given that PTCy-mediated tolerance relies heavily on the rapid functional recovery of spared Tregs, identifying the metabolic substrates that fuel this distinct recovery process could offer novel biomarkers for survival in the modern transplant era.

Because overall survival after HSCT reflects a mixture of relapse-related mortality and non-relapse mortality (NRM), biomarker associations with overall survival can be difficult to interpret without considering the underlying event type. In particular, a marker that tracks early organ dysfunction or systemic inflammation may appear to “predict” overall survival while primarily associating with NRM; conversely, markers related to residual leukemia, treatment resistance, or immune escape may preferentially associate with relapse-related mortality. For this reason, complementary cause-specific and competing-risk analyses can help distinguish whether a candidate marker is more consistent with relapse biology, treatment toxicity, or inflammatory complications, rather than overall prognosis alone. In this context, a clinically useful biomarker should provide incremental prognostic information beyond established transplant and disease risk variables, rather than merely correlate with outcomes in unadjusted analyses [6]. Because clinical variables alone fail to capture this complex biological interplay, there is a need for high-dimensional biomarkers that can read out the systemic physiological state.

Several studies have explored diverse biomarkers (genetic, proteomic, and cellular) to predict post-transplant outcomes, and metabolites are an emerging under-investigated class of biomarkers [6,7]. Metabolites, small molecules serving as both fuels and signalling messengers in immune and cancer biology, can regulate or reflect immune function, inflammation, and even cancer progression [8]. Conceptually, the circulating metabolome provides a proximal, integrated readout of phenotype, capturing the downstream effects of cellular metabolism alongside exposures common in HSCT (e.g., conditioning-related tissue injury, organ dysfunction, infection, medications, and nutritional intake). This breadth is attractive for biomarker discovery but also introduces risk from confounding, emphasizing the need to interpret associations in the context of timing, intercurrent clinical events, and pre-analytical variables. Consistent with this, prior work has reported distinct metabolic patterns associated with transplant complications, including acute GvHD and broader post-transplant outcomes [9,10,11], supporting the plausibility of metabolite-based risk models. For example, increased indoleamine 2,3-dioxygenase (IDO) and the resulting rise in kynurenine [12] have been linked to poorer survival and greater GvHD risk in allogeneic transplant recipients [13]. Similarly, lactate, an end-product of glycolytic metabolism that accumulates under hypoxia or high cell turnover, has immunomodulatory effects. High lactate concentrations in the tumour or tissue microenvironment can directly inhibit cytotoxic T-lymphocyte and NK cell functions, while fostering an immunosuppressive milieu that promotes regulatory immune cells and cancer cell survival [14]. Taken together, these data suggest that systemic metabolic cues may decisively influence the graft-versus-host and graft-versus-leukemia balance. Nevertheless, metabolite concentrations are context-sensitive and may vary with peri-transplant exposures (including conditioning-related tissue injury, organ dysfunction, infection, medications, and nutritional intake), which highlights the importance of transparent reporting of sampling timing and pre-analytical handling to support reproducibility. To facilitate replication across centres and platforms, reporting frameworks such as the Metabolomics Standards Initiative recommend minimum information standards for sample preparation, analytical methods, quality control, and metabolite identification, which is particularly relevant when candidate signals are proposed for future validation and clinical translation [15]. In addition, the high-dimensional nature of metabolomics means that robust multiple-testing control and independent validation are central to distinguishing true biological signals from false positives, particularly when sampling time-points are heterogeneous.

In this study, we hypothesized that levels of specific metabolites in patient plasma could correlate with subsequent transplant outcomes. We conducted a retrospective analysis of a single-centre cohort of 63 adult patients with acute myeloid leukemia (AML) who underwent HSCT to systematically assess the relationships between metabolite levels and key outcomes. Our primary focus was overall survival and cause-specific mortality (relapse-related mortality and non-relapse mortality), with descriptive reporting of GvHD incidence as part of the overall clinical context.

## 2. Materials and Methods

### 2.1. Study Design and Ethical Considerations

This single-centre, retrospective study was conducted at Princess Margaret Cancer Centre, Toronto, Canada, following approval by the Research Ethics Board (CAPCR no. 20-5031) and in accordance with the Declaration of Helsinki. All patients provided informed consent for transplantation and data collection. Clinical management was not influenced by study participation, and clinical variables were abstracted from institutional records for analysis in a coded dataset.

### 2.2. Patient Enrollment and Eligibility Criteria

A total of 63 adult patients with AML who underwent HSCT between March 2016 and April 2021 and had available biobank EDTA plasma samples for at least one of the three sampling statuses (diagnosis, pre-transplant, post-transplant) were included. HSCT ‘day 0’ was defined as the date of stem cell infusion, and all sampling and clinical time-points were referenced to this date. Patients were excluded if they carried a non-AML diagnosis. Collected baseline data included patient age, donor age, donor/recipient sex matching, cytomegalovirus (CMV) serostatus, and history of GvHD. Conditioning regimens, donor source (matched related, matched unrelated), and graft types were recorded. Clinical follow-up continued until death or last known contact (Table 1). Comorbidities were assessed using the hematopoietic stem cell transplantation-comorbidity index (HCT-CI) [16].

### 2.3. GvHD Prophylaxis

Beginning in 2018, our institutional GVHD prophylaxis protocol transitioned to a predominantly PTCy-based platform. Most patients in the study period received PTCy, although alternative regimens were utilized in select cases according to institutional practice (Supplementary Table S1). Our institutional practice at the time was to use ATG-PTCy-CSA if the donors were haploidentical, matched unrelated, or mismatched unrelated, whereas PTCy-CSA-MMF was given for matched related donor transplants. ATG-CSA-MTX was given to those with a cardiac ejection fraction below 50%. ATG could be dropped from the conditioning if the donor was matched related.

### 2.4. Sample Collection and Metabolite Measurement

A total of 263 peripheral blood specimens were drawn in ethylenediaminetetraacetic acid (EDTA) at various time points before and after transplant. Key pre-analytical and clinical-context metadata (including fasting status, time of day, time from venepuncture to processing, storage duration, freeze–thaw history, proximity to transfusion, total parenteral nutrition, corticosteroids, and intercurrent infection) were not systematically captured in the biobank, which may introduce non-biological variability in metabolite levels and limit adjustment for confounding. Across 63 patients, blood samples were drawn a median of 16 days before transplant (range 5–93) and 83 days after transplant (range 20–938). Each patient contributed a median of 1 pre-transplant sample (range 0–2) and 2 post-transplant samples (range 1–12). Samples were centrifuged, and plasma aliquots were stored at −80 °C until batch analysis. Fifty microliters of plasma were collected, flash-frozen in liquid nitrogen vapour, and stored at −80 °C. Metabolomics analyses were performed at the University of Colorado School of Medicine Metabolomics Core by mass spectrometry. Plasma specimens were thawed on ice, then a 20 μL aliquot was diluted with 480 μL of chilled extraction solution (methanol:acetonitrile:water (5:3:2 *v*/*v*)). After vortexing for 30 min at 4 °C, samples were centrifuged at 12,000× *g* for 10 min at 4 °C, and supernatants were isolated for metabolomics analyses. Following randomization of samples, metabolites from 10 μL of sample extract were separated on a Kinetex C18 column (1.7 μm, 150 × 2.1 mm (Phenomenex, Torrance, CA, USA)). Phases utilized for positive ion mode were phase A: water, 0.1% formic acid; phase B: acetonitrile, 0.1% formic acid; negative ion mode—phase A: 1 mM NH_4_OAc 95:5 water:acetonitrile; phase B: 1 mM NH_4_OAc 95:5 acetonitrile:water) via an ultra-high pressure chromatograph (UHPLC, Vanquish, Thermo Fisher, Waltham, MA, USA). The UHPLC was coupled to a high-resolution quadrupole Orbitrap run in both polarity modes via separate runs (Q Exactive, Thermo Fisher) at 70,000 resolution (at *m*/*z* 200), and metabolites were separated through a 5 min gradient [17] with the phases described above. For annotation and peak integration, the raw files (.raw) were converted to .mzXML using RawConverter, then analyzed using Maven alongside the KEGG database and an in-house standard library as previously described [17,18]. Where isobaric features were not chromatographically resolved (e.g., leucine/isoleucine; mannitol/sorbitol), these are reported as combined features as reflected in Section 3.

### 2.5. Data Analysis

Survival analyses incorporated Cox proportional hazards models to assess overall survival (OS), while cause-specific Cox models differentiated between relapse-related mortality and non-relapse mortality (NRM). Cause-specific Cox models were used to estimate cause-specific hazard ratios for relapse-related mortality and NRM. Relapse-free and overall survival Kaplan–Meier curves were generated for visualization. The association between recipient and donor characteristics (age, gender, CMV serostatus) and graft-versus-host disease (acute [aGvHD] and chronic [cGvHD]) was evaluated using Cox proportional hazards analysis. GvHD was incorporated as a time-dependent variable in models to capture its dynamic emergence post-transplant. Relapse was treated as a competing risk for NRM. Competing-risk analyses were performed using Fine and Gray’s subdistribution hazards model, which estimates cumulative incidence while accounting for relapse as the competing event for NRM; subdistribution hazard ratios and 95% confidence intervals are reported [19]. Where hazard ratios are reported, they refer to cause-specific hazards unless explicitly stated otherwise.

Metabolites were transformed by logarithm and examined individually as continuous predictors at three statuses: pre-transplant, diagnosis, and post-transplant, respectively, using a Cox proportional hazard model (or cause-specific model, as appropriate) including the associated covariates of donor age, recipient age, and their interaction. Metabolite associations with relapse-related mortality and NRM were evaluated using cause-specific Cox proportional hazard regression models. Associations among metabolites were explored via hierarchical clustering and Pearson correlation and visualized by heatmaps and a correlation matrix. The association of metabolites with relapse-related mortality and non-relapse mortality (NRM) was evaluated by the cause-specific Cox proportional hazard regression model, in which relapse is the competing risk for NRM. We controlled for multiple testing using false discovery rate (FDR) adjustment and used an exploratory discovery threshold of FDR < 0.25, consistent with a hypothesis-generating biomarker nomination framework.

### 2.6. Outcome Measures

The primary endpoint was OS, defined as time from stem cell infusion to death from any cause or last follow-up. Secondary endpoints included relapse-free survival (RFS) and NRM. Relapse was confirmed by morphologic or molecular criteria, while NRM encompassed death in the absence of relapse. Cause-specific Cox models were used to estimate cause-specific hazard ratios for relapse-related mortality and NRM; unless explicitly stated otherwise, reported hazard ratios refer to cause-specific hazards. Acute GvHD and overall/moderate-severe chronic GvHD were classified using the Mount Sinai aGvHD International Consortium criteria [20] and the National Institutes of Health 2014 Consensus criteria [21], respectively.

### 2.7. Data Presentation

Unless otherwise specified, continuous data are reported as mean ± standard deviation or median with interquartile range, and hazard ratios are presented with 95% confidence intervals.

## 3. Results

### 3.1. Overview of Patient Cohort and Baseline Characteristics

**Figure 1:**
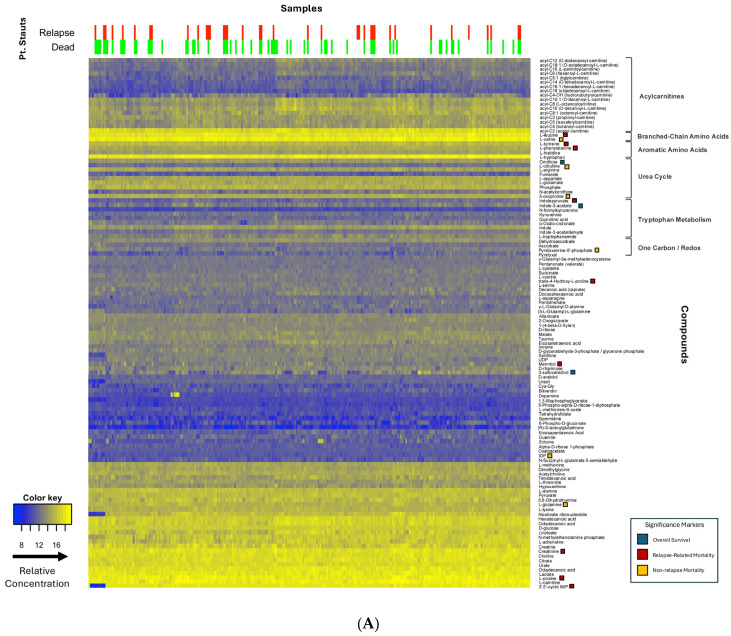

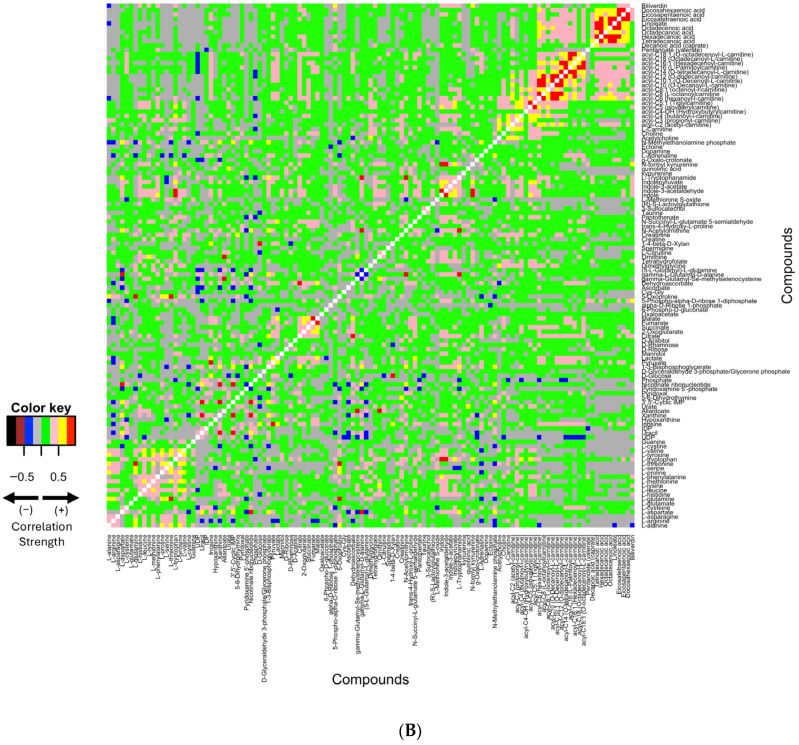
(**A**) Heatmap of metabolite concentrations across plasma samples from 63 AML patients collected at diagnosis, pre-transplant, and post-transplant timepoints (263 total samples). Metabolite levels were log_2_-transformed and z-score normalized prior to hierarchical clustering (Ward’s linkage, Euclidean distance). Columns represent individual patient samples; rows represent metabolites. The colour scale (blue–yellow) indicates relative metabolite abundance, with yellow indicating higher and blue indicating lower levels. Annotation bars above the heatmap indicate relapse status (red = relapsed, green = non-relapsed) and vital status (red = deceased, green = alive). Coloured squares along the right margin denote metabolites reaching the prespecified discovery threshold (FDR < 0.25) in multivariable models: blue = overall survival; red = relapse-related mortality; orange = non-relapse mortality (see Table 2 for full results). Brackets indicate functional metabolite groupings. (**B**) Pairwise Pearson correlation matrix among measured metabolites. The colour scale ranges from −0.5 (blue, negative correlation) through green (near-zero correlation) to 0.5 (red, positive correlation). Metabolites are ordered by hierarchical clustering to highlight correlated modules.

The final study cohort included 63 AML patients who underwent HSCT between March 2016 and April 2021 at our centre, met the inclusion criteria, and comprised the final analytic cohort. Baseline patient demographics and clinical characteristics, including patient and donor ages, gender distribution, and CMV status, are summarized in Table 1. A heatmap of metabolites and a patient-metabolite correlation matrix are presented in Figure 1.

**Table 2:** Metabolites significantly associated with transplant outcomes.

Time-Point	Outcome	Compound	Coefficient	HR	*p* Value	FDR
Diagnosis	Overall survival	No metabolites correlated				
	Relapse-related mortality	No metabolites correlated				
	Non-relapse mortality	No metabolites correlated				
Pre-transplant	Overall survival	No metabolites correlated				
	Relapse-related mortality	L-Phenylalanine	3.280	26.585	0.001	0.111
		L-Leucine	2.375	10.755	0.001	0.111
		Indolepyruvate	2.038	7.676	0.005	0.171
		Creatinine	2.630	13.874	0.005	0.171
		trans-4-Hydroxy-L-proline	−2.290	0.101	0.003	0.171
		Mannitol	1.100	3.005	0.013	0.209
		L-Tyrosine	1.927	6.870	0.013	0.209
		3′,5′-Cyclic-IMP	3.087	21.919	0.013	0.209
		L-Proline	1.964	7.128	0.012	0.209
	Non-relapse mortality	L-Valine	3.197	24.454	0.003	0.171
		IDP	−2.396	0.091	0.004	0.171
		Pyridoxamine-5′-phosphate	−1.161	0.313	0.006	0.171
		L-Citrulline	3.019	20.478	0.008	0.204
		5-Oxoproline	2.465	11.766	0.010	0.209
		L-Glutamine	2.163	8.701	0.013	0.209
		Ornithine	−2.757	0.063	0.001	0.089
	Overall survival	3-Sulfocatechol	−0.528	0.590	0.004	0.226
Post-transplant		Indole-3-acetate	−1.024	0.359	0.006	0.240
	Relapse-related mortality	No metabolites correlated				
	Non-relapse mortality	No metabolites correlated				

The table lists all significant metabolites (*p* < 0.05, FDR < 0.25) at three clinical time-points: diagnosis, pre-transplant, and post-transplant. Results are stratified by outcome. Abbreviations: HR, hazard ratio; FDR, false discovery rate; IDP, inosine diphosphate; 3′,5′-Cyclic-IMP, 3′,5′-cyclic inosine monophosphate.

### 3.2. Primary Outcomes

**Figure 2:**
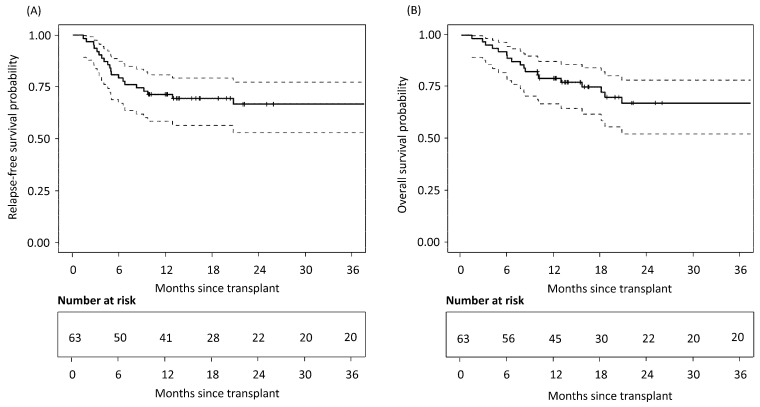
Kaplan–Meier survival curves illustrating relapse-free survival (**A**) and overall survival (**B**) following allogeneic stem cell transplantation. Dashed lines indicate 95% confidence intervals. N = 63.

Kaplan–Meier analyses characterized survival outcomes following HSCT. Survival at 2 years was 67.3% (95% CI: 52.4–78.4), and relapse-free survival at 2 years was 66.8% (95% CI: 53.0–77.4%), Figure 2. Cumulative incidence estimates for key transplant events (including relapse and non-relapse mortality, and acute and chronic GvHD) are summarized in Table 1.

### 3.3. Clinical Covariate Models Informing Adjustment

In Cox proportional hazards modelling for overall survival, donor age (HR = 0.811, *p* = 0.032) and the donor age by recipient age interaction (HR = 1.003, *p* = 0.039) were significant predictors (Supplementary Table S2), while recipient age showed a trend (HR = 0.929, *p* = 0.076). Given the significant donor age by recipient age interaction, metabolite associations with overall survival are presented as multivariable estimates adjusted for these covariates rather than univariable screening results. In cause-specific multivariate analyses, younger donor age significantly correlated with increased relapse risk (HR = 0.946, *p* = 0.016), whereas older recipient age at the time of transplantation was significantly associated with higher non-relapse mortality (HR = 1.060, *p* = 0.041) (Supplementary Table S3).

### 3.4. Metabolite Associations by Outcome

#### 3.4.1. Overall Survival

**Figure 3:**
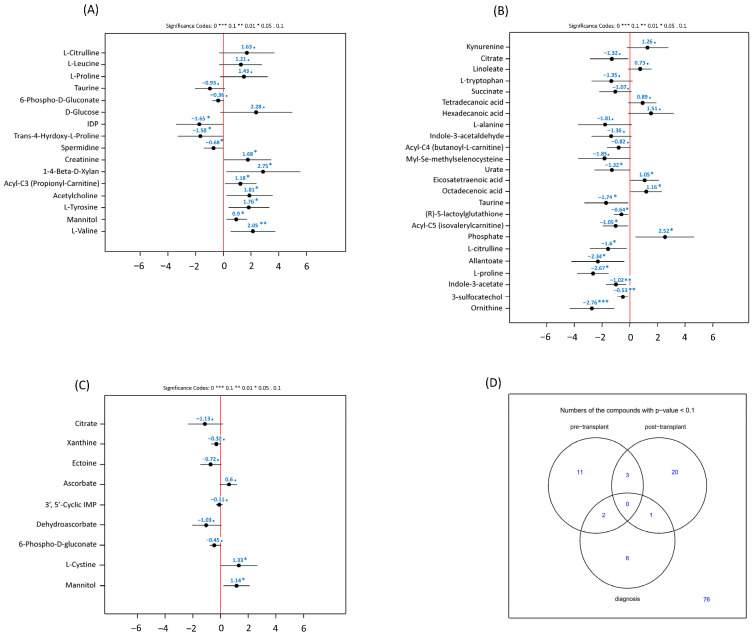
Metabolites associated with overall survival (OS). Forest plots depicting hazard ratios (HR) and 95% confidence intervals (CI) for metabolites significantly associated with overall survival post-transplant. Panels represent (**A**) pre-transplant, (**B**) post-transplant, and (**C**) diagnosis metabolites. Panel (**D**) shows a Venn diagram summarizing overlaps between these significant metabolites. (*** *p* < 0.001, ** *p* < 0.01, * *p* < 0.05). N = 63. Number of patients and samples per period: diagnosis (60 patients, 61 samples), pre-transplant (62 patients, 64 samples), and post-transplant (63 patients, 138 samples).

Metabolites significantly associated with overall survival are summarized by sampling status in Figure 3A–C, with overlap across diagnosis, pre-transplant, and post-transplant findings shown in Figure 3D. Using the prespecified discovery threshold (FDR < 0.25), no metabolites measured at diagnosis were associated with overall survival. Several pre-transplant metabolites showed nominal associations with reduced overall survival; however, none met the prespecified FDR threshold after multiple-testing adjustment (FDR ≥ 0.25, Supplementary Table S4).

Higher post-transplant ornithine (HR 0.06; *p* = 0.001; FDR = 0.089), 3-sulfocatechol (HR 0.590; *p* = 0.004; FDR = 0.226), and indole-3-acetate (HR 0.359; *p* = 0.006; FDR = 0.240) were associated with improved OS (lower hazard of death) (Table 2) in models adjusting for donor age, recipient age, and their interaction. No metabolite was associated with overall survival across all three timepoints (Figure 3D). However, three metabolites demonstrated associations with overall survival (*p* < 0.1) at both the pre-transplant and post-transplant timepoints: L-citrulline, taurine, and L-proline. Notably, the direction of association reversed for two of the three. Higher pre-transplant L-citrulline and L-proline were associated with worse survival (coefficients +1.63 and +1.43, respectively), whereas higher post-transplant levels of the same metabolites were associated with improved survival (L-citrulline coefficient −2.34, *p* < 0.01; L-proline coefficient −1.02, *p* < 0.01). In contrast, taurine was associated with improved survival at both timepoints (pre-transplant coefficient −0.93; post-transplant coefficient −0.64, *p* < 0.05). These cross-timepoint associations did not meet the prespecified FDR threshold of 0.25 at both timepoints simultaneously and are therefore reported as exploratory observations (Figure 3D).

#### 3.4.2. Relapse-Related Mortality

**Figure 4:**
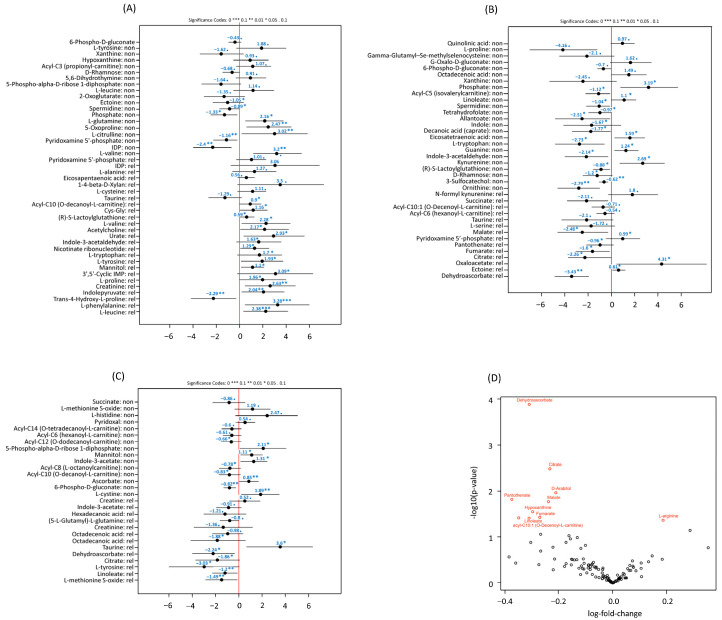
(**A**) Pre-transplant metabolites predictive of relapse and non-relapse mortality. Forest plots demonstrating the effect sizes (log hazard ratio) and 95% CIs for pre-transplant metabolites associated with cause-specific mortality (relapse vs. non-relapse). For visualization, metabolites meeting unadjusted thresholds are displayed, and stars indicate unadjusted *p*-values; prespecified discovery is based on FDR < 0.25 as reported in the Supplementary Tables; metabolites are labelled accordingly for relapse (“rel”) and non-relapse mortality (“non”). (*** *p* < 0.001, ** *p* < 0.01, * *p* < 0.05). (**B**) Post-transplant metabolites predictive of relapse and non-relapse mortality. Forest plots illustrating significant post-transplant metabolites predictive of relapse-related and non-relapse mortality. Positive effect sizes indicate higher mortality risk, while negative values suggest protective effects. Statistical significance levels are indicated with standard symbols (*** *p* < 0.001, ** *p* < 0.01, * *p* < 0.05). (**C**) Diagnostic metabolites predictive of relapse and non-relapse mortality. Forest plots showing diagnostic metabolites significantly associated with subsequent relapse or non-relapse mortality post HSCT. Effect sizes (log hazard ratios) with 95% confidence intervals are shown. (*** *p* < 0.001, ** *p* < 0.01, * *p* < 0.05). (**D**) Volcano plot of metabolite differences between patients who relapsed and those who did not (N = 63). Each point represents one of the quantified metabolites. The horizontal axis represents the relative difference in average metabolite level between the relapse and non-relapse groups on a base-2 logarithmic scale. Points to the right of zero were more abundant in relapse samples, whereas points to the left were less abundant. The ordinate shows the negative base-10 logarithm of the *p*-value, so each unit higher corresponds to a ten-fold smaller *p*-value from the mixed-effects model described in Methods. Metabolites with *p* < 0.05 are coloured red and annotated with their common names. Unfilled black circles represent metabolites that did not reach significance.

Given that OS is a composite of competing events, we next utilized cause-specific models to dissect whether these metabolic signals were driving relapse or toxicity. While post-transplant metabolites are primarily associated with overall survival, we sought to determine if pre-transplant metabolic states could specifically stratify the risk of leukemic relapse. At diagnosis, no metabolites met the prespecified threshold for transplant outcomes, including relapse-related mortality. In pre-transplant samples (Supplementary Table S5; Figure 4A), phenylalanine (HR 26.59; *p* = 0.001; FDR = 0.111), leucine/isoleucine (HR 10.76; *p* = 0.001; FDR = 0.111), indolepyruvate (HR 7.68; *p* = 0.005; FDR = 0.171), creatinine (HR 13.87; *p* = 0.005; FDR = 0.171), mannitol/sorbitol (HR 3.01; *p* = 0.013; FDR = 0.209), tyrosine (HR 6.87; *p* = 0.013; FDR = 0.209), 3′,5′-cyclic-IMP (HR 21.92; *p* = 0.013; FDR = 0.209), and proline (HR 7.13; *p* = 0.012; FDR = 0.209) were associated with an increased risk of relapse-related mortality, whereas trans-4-hydroxy-L-proline (HR 0.10; *p* = 0.003; FDR = 0.171) was protective.

In post-transplant analyses, several metabolites demonstrated associations with relapse-related mortality on unadjusted testing; however, none met the prespecified FDR threshold of 0.25 (Supplementary Table S6; Figure 4B). None of the metabolites measured at diagnosis met the FDR threshold for relapse-related mortality (Supplementary Table S7).

#### 3.4.3. Non-Relapse Related Mortality

At diagnosis, no metabolites were associated with non-relapse mortality (Table 2). In pre-transplant analyses, significant predictors for non-relapse mortality included valine (HR 24.45; *p* = 0.003; FDR = 0.171), 5-oxoproline (HR = 11.766; *p* = 0.010; FDR = 0.209), glutamine (HR = 8.701; *p* = 0.013, FDR = 0.209) and citrulline (HR 20.48; *p* = 0.008; FDR = 0.204), as well as IDP (HR 0.09; *p* = 0.004; FDR = 0.171) and pyridoxamine-5′-phosphate (HR 0.313; *p* = 0.006; FDR = 0.171) (Supplementary Table S5; Figure 4A). In post-transplant analyses, metabolites associated with non-relapse mortality did not meet the FDR threshold of 0.25 (Supplementary Table S6; Figure 4B).

### 3.5. Mixed Effects Modelling

In analyses utilizing linear mixed-effects models to identify differential metabolite levels between relapse and non-relapse patients, dehydroascorbate (coef = −0.31, *p* = 0.00013, FDR = 0.02) and citrate (coef = −0.23, *p* = 0.0033, FDR = 0.19) were the most significantly altered metabolites (Supplementary Table S8; Figure 4D), and were reduced in patients who experienced relapse. Other metabolites, including arabitol (coef = −0.21; *p* = 0.011), pantothenate (coef = −0.38; *p* = 0.015), and malate (coef = −0.24; *p* = 0.017), were also altered in relapsed patients; however, these associations did not maintain significance after adjusting for false discovery rate (FDR ≥ 0.25). Overall, these findings underscore dehydroascorbate as a potentially robust metabolic biomarker for relapse risk in AML patients post-transplantation.

## 4. Discussion

In this retrospective, single-centre, biobank-based study, we identified specific plasma metabolites that were associated with clinical outcomes following allogeneic HSCT in adults with AML. In models adjusting for donor age, recipient age, and their interaction, higher post-transplant ornithine, 3-sulfocatechol, and indole-3-acetate (each HR < 1) were associated with improved overall survival (lower hazard of death). Pre-transplant leucine, phenylalanine, trans-4-hydroxy-L-proline, indolepyruvate, and creatinine were independently associated with relapse-related mortality, while significant pre-transplant predictors for non-relapse mortality included valine, IDP, pyridoxamine-5′-phosphate, citrulline, 5-oxoproline, and glutamine (Table 2). Mixed-effects modelling further identified lower dehydroascorbate and citrate in patients who relapsed. Given the modest cohort size, limited covariate adjustment, and heterogeneity of sampling timing, these associations should be interpreted as candidate biomarker signals for validation rather than prescriptive risk thresholds or treatment guides.

The association between preserved post-transplant ornithine levels and improved overall survival is particularly relevant in our cohort, where 81% of patients received PTCy-based prophylaxis. PTCy efficacy is biologically predicated on the preferential survival and subsequent expansion of regulatory T-cells (Tregs) to enforce immune tolerance [4], and ornithine serves as the obligate precursor for polyamine synthesis (putrescine, spermidine, and spermine) via ornithine decarboxylase. Recent data demonstrate that intracellular spermidine is essential for Treg lineage commitment and phenotypic stability [22]. Accordingly, lower ornithine levels in patients with poor outcomes may reflect systemic substrate limitation that compromises polyamine synthesis and, in turn, diminishes the Treg reconstitution central to the PTCy platform, suggesting that adequate circulating ornithine may be a requisite ‘metabolic fuel’ for PTCy-based immune tolerance [22]. Polyamines may also remodel anti-cancer immunity through mechanisms such as autophagy, which is necessary for T cell activation, function, and survival [23]. Beyond ornithine, several nominated signals implicate amino-acid metabolism in post-transplant immune homeostasis and relapse biology: elevated pre-transplant leucine/isoleucine (predictive of relapse-related mortality) may reflect metabolic dysregulation linked to aggressive leukemia phenotypes or impaired immune surveillance, while glutamine is required for lymphocyte activation and proliferation and is rapidly consumed during T-cell clonal expansion [24]. Similarly, arginine depletion (via arginase or other pathways) can cause T-cell dysfunction and has been linked to immune suppression in cancer and allogeneic settings [25]. Disruptions to arginine metabolism, favouring conversion to ornithine and citrulline, have also been associated with ageing in humans [26]. Furthermore, L-glutamine and L-citrulline are central to nitrogen metabolism and immune cell proliferation, highlighting their potential impact on immune reconstitution and graft function [27].

Among the metabolites assessed, three, L-citrulline, L-proline, and taurine, were associated with overall survival at both the pre-transplant and post-transplant timepoints (Figure 3D). The directional reversal observed for L-citrulline and L-proline is particularly noteworthy: elevated pre-transplant levels were associated with worse survival, while elevated post-transplant levels were protective. One plausible interpretation is that the biological meaning of these metabolites differs by transplant phase. Pre-transplant elevations in citrulline and proline may reflect residual disease burden, hepatic or renal dysfunction, or a catabolic state associated with prior chemotherapy, all of which are established adverse prognostic features [28]. In contrast, post-transplant citrulline is an established marker of enterocyte mass and mucosal integrity, and its recovery following conditioning-related mucosal injury has been associated with lower transplant-related toxicity and improved nutritional status [29]. Similarly, post-transplant proline recovery may reflect restoration of collagen synthesis and tissue repair. This context-dependent reversal illustrates that the same metabolite can be a marker of harm in one setting and of recovery in another, and underscores the importance of timepoint-specific interpretation in metabolomic studies.

Taurine, by contrast, was protective at both timepoints. Taurine is a semi-essential amino acid with established antioxidant, anti-inflammatory, and cytoprotective properties, and has been implicated in the protection of tissues exposed to oxidative stress, including the intestinal epithelium and the liver [30]. Its consistent protective association across transplant phases may reflect a systemic anti-inflammatory milieu that is favourable both before and after transplantation, and is biologically coherent with the role of oxidative stress in conditioning-related tissue injury and post-transplant organ toxicity. These cross-timepoint findings should be considered exploratory, as they were identified at *p* < 0.1 and did not uniformly meet the FDR < 0.25 threshold at both timepoints, but they nominate citrulline, proline, and taurine as metabolites of particular interest for prospective validation with protocol-defined sampling.

Branched-chain amino acids such as valine have been implicated in hematopoietic stem cell maintenance and immune reconstitution: in murine models, dietary valine restriction can reversibly deplete hematopoietic stem cells from the bone marrow niche [31]. Together with evidence that elevated branched-chain amino acids may correlate with increased leukemia aggressiveness and relapse risk, potentially via pathways supporting tumour cell proliferation and resistance to apoptosis [25], these findings support the concept that the systemic metabolic state could shape graft-versus-host reactions and graft-versus-leukemia effects, influencing clinical outcomes. Importantly, because we did not control for total parenteral nutrition administration, the significance of the valine findings should be interpreted cautiously, and we cannot exclude that elevated branched-chain amino acids (including valine) partially reflect parenteral supplementation during severe illness (e.g., GvHD-associated anorexia) rather than intrinsic drivers of disease biology.

Mixed-effects modelling identified dehydroascorbate as significantly lower in patients experiencing relapse (coef = −0.31; *p* = 0.00013; FDR = 0.02), with citrate also reduced (coef = −0.23; *p* = 0.0033; FDR = 0.19), nominating dehydroascorbate (DHA; oxidized vitamin C) as a candidate relapse-risk biomarker for future validation studies. DHA has roles in redox balance, immune modulation, and apoptosis regulation, mechanisms relevant to post-transplant leukemia control. The association between lower DHA and relapse is mechanistically compelling, given that ascorbate is a cofactor for Ten-eleven translocation (TET) methylcytosine dioxygenases; TET2 loss-of-function mutations are prevalent in AML and contribute to a hypermethylated, leukemogenic epigenetic landscape. Agathocleous et al. [32] demonstrated that ascorbate depletion in hematopoietic stem cells accelerates leukemogenesis, while repletion restricts disease progression, raising the possibility that lower circulating DHA reflects systemic depletion permissive of residual leukemic clone survival. Other nominated metabolites also provide biologically plausible anchors: indole-3-acetate and indolepyruvate (tryptophan catabolites) suggest altered tryptophan pathways influencing immune tolerance and inflammatory responses [12]. Creatinine reflects renal function [33] and muscle metabolism, possibly implicating tissue turnover and organ stress post-transplant [8], and thus may be a proxy for kidney injury, a known transplant risk factor. The identification of creatinine acts as a biological positive control, supporting that our platform captures clinically relevant organ dysfunction; the co-identification of indoles and DHA suggests that metabolomic profiling may provide prognostic information beyond standard biochemical indices. Pyridoxamine-5′-phosphate, a vitamin B6 derivative, also plays critical roles in amino acid metabolism and antioxidative defence, potentially influencing recovery and immune regulation post-transplant [24].

Pathway-level findings were consistent with these metabolite-level associations. Pre-transplant arginine metabolism (involving glutamine and citrulline) plays essential roles in immune cell function, tissue repair, and nitric oxide production [34]. Post-transplant, significant alterations in tryptophan metabolism, particularly involving kynurenine, may indicate immune dysregulation, potentially contributing to increased infection risk and associated mortality due to its immunosuppressive properties [9,13]. Pre-transplant disruptions in phenylalanine, tyrosine, and tryptophan and their pathways are notable due to their roles in inflammation and innate immune responses, potentially fostering an aggressive leukemic environment predisposing to relapse.

Weight loss of ≥5% is observed in a considerable number of HSCT recipients in the first month post-HSCT [35] and is linked to reductions in circulating amino-acid pools, with total plasma amino-acid concentrations on day +7 falling along with inflammatory markers, and plausibly contributing to some metabolomic shifts [36]. However, targeted metabolic comparisons of patients given adequate enteral versus mainly parenteral nutrition show that conditioning and transplant-related biology, rather than nutrition route or calorie adequacy, drive the majority of early post-transplant metabolic variance, suggesting that weight-loss-related effects explain only a limited part of our findings [37].

Prior HSCT metabolomics studies provide important context. Landfried et al. showed that higher indoleamine 2,3-dioxygenase (IDO) activity and kynurenine levels after HSCT were associated with a greater risk of acute GvHD and non-relapse mortality [13], potentially reflecting intense inflammatory stimuli accompanying severe GvHD. Michonneau et al. reported that acute GvHD was associated with distinct host- and microbiota-derived metabolite changes, including lower kynurenine and other tryptophan derivatives at the onset of GvHD, consistent with reduced immunoregulatory metabolites permitting unchecked alloreactive T-cell activity [9]. This is also supported by previous work showing that absent oral intake was associated with a significantly higher incidence of GvHD [38]. While we analyzed plasma metabolites, signals such as indole-3-acetate (IAA) are almost exclusively derived from the gut microbiota. IAA is an AhR ligand important for intestinal barrier integrity and dampening GvHD inflammation [39], and our finding that higher post-transplant IAA predicts better survival is in keeping with a model in which a preserved microbiome protects against transplant toxicity. Future studies should pair plasma metabolomics with stool metagenomics to confirm the bacterial origin of these protective indoles. Beyond GvHD, pre-transplant metabolomic signatures have been associated with later severe acute GvHD or early NRM [11], and chronic GvHD and long-term immunosuppression have been linked to altered plasma metabolite patterns [10]. Overall, concordance with prior HSCT metabolomics literature supports biological plausibility for the pathways implicated here, while recognizing that the present study remains associative and requires independent validation.

Nevertheless, this study has several limitations. Firstly, our retrospective, single-centre design and relatively modest sample size (n = 63) limit the generalizability and robustness of our conclusions, and findings require validation in larger, prospective cohorts across diverse clinical settings. Further, the retrospective nature of the study limits our ability to capture dynamic metabolic fluctuations during transplantation and post-transplant recovery. Longitudinal metabolomic analyses would provide more comprehensive insights into metabolic trajectories and their clinical implications.

A further important limitation is the heterogeneity of sample collection. Because samples were drawn from a clinical biobank as part of routine care rather than under a research protocol with pre-specified sampling windows, collection timepoints varied across patients (pre-transplant: median 16 days before transplant, range 5–93; post-transplant: median 83 days, range 20–938; Supplementary Table S1). We categorized samples into three clinically meaningful periods (diagnosis, pre-transplant, post-transplant) and analyzed metabolites within each period separately, which preserves statistical power but introduces within-period variability that may attenuate true associations or introduce confounding from intercurrent clinical events. Restricting analyses to patients with samples at identical protocol-defined timepoints would improve temporal resolution but was not feasible without reducing the cohort below the threshold for meaningful multivariable survival modelling. Additionally, key pre-analytical metadata, including fasting status, time from venepuncture to processing, storage duration, freeze–thaw cycles, and proximity to transfusion, parenteral nutrition, corticosteroids, or intercurrent infection, were not systematically captured in the biobank, which may introduce non-biological variability in metabolite levels and limit adjustment for confounding. Future validation studies should enforce protocolized landmark sampling (e.g., pre-conditioning, Day 0, Day +30, Day +100) with systematic capture of pre-analytical variables and intercurrent events at each blood draw, to distinguish transient recovery-phase signals from stable prognostic markers and to reduce confounding by peri-transplant exposures.

Our study was designed with overall survival and cause-specific mortality (relapse-related and non-relapse) as pre-specified endpoints, as these are the outcomes most directly relevant to patient prognosis after HSCT. We did not systematically assess metabolite associations with individual transplant complications such as GvHD subtypes, infections, graft rejection, or organ toxicities. Such analyses would require substantially larger cohorts with dedicated study designs and appropriate multiplicity control; however, given the potential clinical value of predicting specific complications at defined timepoints, this represents an important direction for future investigation.

Associations near the FDR threshold should be interpreted cautiously due to the potential for type I errors, and several nominated metabolites may act as surrogates of concomitant physiology (e.g., renal or hepatic dysfunction, infection, or nutritional intake) rather than direct mediators of outcome. Some metabolite differences may represent early manifestations of complications rather than antecedent risk, and this potential for reverse causality should be considered when interpreting pathway-level hypotheses. If robust, such signals could be incorporated into post-transplant surveillance pathways to trigger earlier investigations, targeted supportive care, or trial enrolment for pre-emptive strategies, but not in isolation to escalate immunosuppression or other high-risk interventions.

While our study includes a small subset of patients receiving conventional prophylaxis (19%), the predominance of PTCy-treated patients allows these findings to be interpreted as a specific characterization of the metabolic landscape in the modern haplo-identical and matched-donor PTCy era, rather than a direct comparison between prophylactic regimens.

In conclusion, our study supports the feasibility of using plasma metabolomics to nominate candidate biomarkers associated with survival and cause-specific outcomes after allogeneic HSCT in AML. Future work should confirm these findings in larger, multicentre prospective cohorts and explore mechanisms using integrative multi-omics approaches, including testing whether metabolite-informed models improve risk discrimination beyond standard clinical covariates and remain robust across platforms and centres.

## Figures and Tables

**Table 1:** Baseline patient demographics and clinical characteristics.

Characteristic	Value
Patient age (years), median (range)	59 (19–71)
Sex, N (%) Male/Female	37/26 (59%/41%)
Karnofsky performance status, N (%)	
70–80	7 (11%)
90–100	55 (87%)
Missing	1 (2%)
Patient CMV (recipient/donor), N (%)	
Neg/Neg	7 (11%)
Neg/Pos	2 (3%)
Pos/Pos	24 (38%)
Pos/Neg	30 (48%)
Cytogenetics, N (%)	
Favourable	4 (6%)
Intermediate	34 (54%)
Adverse	16 (25%)
Unknown	9 (14%)
Disease status at transplant (CR1/CR2), N (%)	55/8 (87%/13%)
Disease risk index, N (%)	
0 (Low)	2 (3%)
1 (Intermediate)	48 (76%)
2 (High)	12 (19%)
3 (Very high)	1 (2%)
Comorbidity index, N (%)	
HCT-CI = 0	10 (16%)
HCT-CI = 1 or 2	26 (41%)
HCT-CI= or >3	25 (40%)
Missing	2 (3%)
Donor age (years) median (range)	30 (11–74)
Donor type, N (%)	
Matched sibling donor	13 (21%)
Matched unrelated donor (MUD)	41 (65%)
Haploidentical donor (Haplo)	9 (14%)
ABO compatibility (match/mismatch), N (%)	28/35 (40%/60%)
Stem cell source (PB/BM), N (%)	62/1 (98%/2%)
CD34 dose median (range), N (%)	7.08 (2.47–18.39)
Conditioning regimen (MAC/RIC), N (%)	15/48 (24%/76%)
GvHD prophylaxis, N (%)	
ATG-CSA-MTX	9 (14%)
ATG-PTCy-CSA	46 (73%)
CSA-MTX	3 (5%)
PTCy-CSA-MMF	5 (8%)
Median follow-up (days) median (range)	365 (41–1357)
Overall survival at 2 years (95% CI)	67% (52–78)
Relapse-free survival at 2 years (95% CI)	67% (53–77)
Cumulative incidence of aGvHD at 6 months (95% CI)	43% (30–55)
Cumulative incidence of cGvHD at 2 years (95% CI)	34% (17–51)
Cumulative incidence of relapse at 2 years (95% CI)	16% (8–26)
Nonrelapse mortality at 1 year (95% CI)	13% (6–22)

Abbreviations: ABO, blood group system (A, B, and O); aGvHD, acute graft-versus-host disease; ATG, anti-thymocyte globulin; BM, bone marrow; CI, confidence interval; CMV, cytomegalovirus; CR1, first complete remission; CR2, second complete remission; CSA, cyclosporine; cGVHD, chronic graft-versus-host disease; Haplo, haploidentical donor (partially matched donor); HCT-CI, hematopoietic cell transplantation-specific comorbidity index; MAC, myeloablative conditioning; MMF, mycophenolate mofetil; MTX, methotrexate; MUD, matched unrelated donor; PB, peripheral blood; PTCy, post-transplant cyclophosphamide; and RIC, reduced-intensity conditioning.

## Data Availability

Data are available upon request from the authors at their discretion.

## Supplementary Materials

The following supporting information can be downloaded at: https://www.mdpi.com/article/10.3390/hematolrep18020027/s1, Table S1: Patient-level characteristics for each study participant; Table S2: Clinical Covariates Associated with Overall Survival; Table S3: Clinical Covariates Associated with Relapse and Non-Relapse Mortality; Table S4: Significant Metabolites Affecting Overall Survival; Table S5: Pre-transplant Metabolites Predictive of Cause-Specific Mortality; Table S6: Post-transplant Metabolites Predictive of Cause-Specific Mortality; Table S7: Diagnostic Metabolites Predictive of Cause-Specific Mortality; Table S8: Top Compounds Associated with Relapse.
