# Supplementary material for: Metabolomic Signatures of Relapse and Survival in AML Patients Receiving Allogeneic Hematopoietic Stem Cell Transplantation
Source: Hematol Rep. 2026 Apr 7;18(2):27. doi: 10.3390/hematolrep18020027 (PMC13116989; doi:10.3390/hematolrep18020027)
Supplement: Supplementary file 1 [file hematolrep-18-00027-s001.zip › hematolrep-4133767-supplementary.pdf]

Metabolomic Signatures of Relapse and Survival in AML Patients Receiving Allogeneic Hematopoietic Stem Cell Transplantation

Igor Novitzky-Basso [1,2,*], Changjiang Xu [3], Caden Chiarello [1], Julie A. Reisz [4], Angelo D'Alessandro [4], Gary D. Bader [3], Jonas Mattsson [1,2,†] and Courtney Jones [5,†]

Supplementary information

Supplementary Information (PDF) contains eight tables: patient-level characteristics (Table S1), clinical covariates influencing overall survival, relapse and non-relapse mortality (Tables S2 & S3); metabolites measured at diagnosis, pre- and post-transplant associated with overall survival and cause-specific mortality (Tables S4-S7); and a mixed-effects analysis of metabolite differences between relapse and non-relapse patients (Table S8).

Table S1. Patient-level characteristics for each study participant.

UPN	1	2	3	4	5	6	7	8	9
Age at BMT	33	22	69	67	44	61	39	66	62
Gender	Male	Male	Female	Male	Female	Male	Male	Female	Male
Stage	CR2	CR1	CR1	CR1	CR1	CR1	CR1	CR1	CR1
DRI (0-4)	2	1	1	1	1	1	1	1	2
Donor	URD	MRD	Haplo	URD	MRD	URD	MRD	URD	URD
RIC/MAC	RIC	MAC	RIC	RIC	MAC	RIC	MAC	RIC	RIC
GVH Prophylaxis	ATG-PTCy-CSA	PTCy-CSA-MMF	ATG-PTCy-CSA	ATG-PTCy-CSA	CSA-MTX	ATG-PTCy-CSA	PTCy-CSA-MMF	ATG-PTCy-CSA	ATG-CSA-MTX
Graft Source	PB	PB	PB	PB	PB	PB	PB	PB	PB
aGvHD YN	Y	N	N	Y	Y	N	Y	Y	Y
aGVH Grade	grade2			grade1	grade3		grade2	grade2	grade3
cGvHD YN	N	Y	N	N	N	N	Y	N	N
cGVH Grade		moderate					moderate		
Died	N	N	N	N	Y	N	N	Y	Y
Relapsed	Y	N	N	N	N	N	N	Y	N
# of Samples at Diagnosis	0	1	1	1	1	1	1	1	1
Day of Collection Pre-HCT		112	119	117	156	135	533	176	323
# of Samples Pre-HCT	1	1	1	1	1	1	1	1	1

**Days of Collection Pre-HCT (Range)**	50	18	13	14	7	14	20	23	16
**# of Samples Post-HCT**	2	1	3	2	1	1	1	1	1
**Days of Collection Post-HCT (Range)**	43-61	31	70-178	72-100	99	155	191	229	58
**Median Days**	52	31	87	86	99	155	191	229	58

**UPN**	**10**	**11**	**12**	**13**	**14**	**15**	**16**	**17**	**18**
**Age at BMT**	64	59	58	54	52	71	70	42	71
**Gender**	Male	Female	Female	Male	Female	Male	Male	Male	Male
**Stage**	CR2	CR1	CR1	CR1	CR1	CR1	CR1	CR1	CR2
**DRI (0-4)**	1	1	1	1	1	1	2	2	1
**Donor**	URD	MRD	URD	URD	MRD	URD	URD	Haplo	URD
**RIC/MAC**	RIC	MAC	RIC	MAC	MAC	RIC	RIC	RIC	RIC
**GVH Prophylaxis**	CSA-MTX	CSA-MTX	ATG-PTCy-CSA	ATG-PTCy-CSA	PTCy-CSA-MMF	ATG,CSA, MTX	ATG-PTCy-CSA	ATG-PTCy-CSA	ATG-PTCy-CSA
**Graft Source**	PB	PB	PB	PB	PB	PB	PB	PB	PB
**aGvHD YN**	Y	Y	N	N	Y	Y	Y	N	Y
**aGVH Grade**	grade3	grade2			grade2	grade2	grade2		grade2
**cGvHD YN**	Y	N	N	N	Y	N	Y	N	N
**cGVH Grade**	moderate				moderate		mild		

**Died**	N	N	Y	N	N	Y	N	N	N
**Relapsed**	N	N	N	N	N	N	N	N	N
**# of Samples at Diagnosis**	1	1	1	1	1	1	1	1	1
**Day of Collection Pre-HCT**	110	180	154	107	116	196	108	179	414
**# of Samples Pre-HCT**	1	1	1	1	1	1	1	1	1
**Days of Collection Pre-HCT (Range)**	2	33	5	22	14	21	17	48	14
**# of Samples Post-HCT**	2	1	3	1	2	3	3	10	1
**Days of Collection Post-HCT (Range)**	34-62	49	35-101	27	41-55	58-92	81-938	31-906	110
**Median Days**	48	49	56	27	48	76	88	228	110
**UPN**	19	20	21	22	23	24	25	26	27
**Age at BMT**	65	59	56	70	58	69	67	68	65
**Gender**	Male	Male	Male	Female	Male	Male	Male	Male	Female
**Stage**	CR1	CR1	CR1	CR1	CR1	CR1	CR1	CR1	CR1
**DRI (0-4)**	1	1	2	1	1	1	2	1	1
**Donor**	MRD	URD	URD	URD	URD	URD	Haplo	URD	Haplo
**RIC/MAC**	RIC	RIC	RIC	RIC	MAC	RIC	RIC	RIC	RIC

**GVH Prophylaxis**	ATG-PTCy-CSA	ATG-CSA-MTX	ATG-PTCy-CSA	ATG-PTCy-CSA	ATG-PTCy-CSA	ATG-PTCy-CSA	ATG-PTCy-CSA	ATG-CSA-MTX	ATG-PTCy-CSA
**Graft Source**	PB	PB	PB	PB	PB	PB	PB	PB	PB
**aGvHD YN**	N	Y	N	N	Y	N	N	Y	N
**aGVH Grade**		grade2			grade2			grade3	
**cGvHD YN**	Y	Y	N	N	N	N	N	Y	Y
**cGVH Grade**	mild	moderate						moderate	moderate
**Died**	N	Y	Y	N	N	Y	Y	N	N
**Relapsed**	N	Y	Y	N	N	N	N	N	N
**# of Samples at Diagnosis**	1	1	0	1	1	1	1	1	1
**Day of Collection Pre-HCT**	133	177		170	182	131	113	103	245
**# of Samples Pre-HCT**	1	1	1	1	1	1	1	1	1
**Days of Collection Pre-HCT (Range)**	12	29	8	15	14	21	20	14	93
**# of Samples Post-HCT**	1	1	2	3	2	1	1	3	2
**Days of Collection**	125	43	69-83	30-86	30-89	76	58	20-139	67-280

**Post-HCT (Range) Median Days**	125	43	76	58	60	76	58	72	174
**UPN**	**28**	**29**	**30**	**31**	**32**	**33**	**34**	**35**	**36**
**Age at BMT**	35	40	54	30	59	35	71	65	29
**Gender**	Male	Male	Male	Male	Male	Male	Female	Male	Male
**Stage**	CR1	CR1	CR1	CR1	CR1	CR1	CR1	CR1	CR2
**DRI (0-4)**	0	1	1	1	1	1	1	2	1
**Donor**	URD	URD	URD	Haplo	URD	URD	URD	URD	URD
**RIC/MAC**	MAC	RIC	RIC	RIC	RIC	RIC	RIC	RIC	RIC
**GVH Prophylaxis**	ATG-PTCy-CSA	ATG-CSA-MTX	ATG-PTCy-CSA	ATG-PTCy-CSA	ATG-PTCy-CSA	ATG-PTCy-CSA	ATG-PTCy-CSA	ATG-CSA-MTX	ATG-PTCy-CSA
**Graft Source**	PB	PB	PB	PB	PB	PB	PB	PB	PB
**aGvHD YN**	N	N	N	Y	N	N	N	Y	N
**aGVH Grade**				grade2				grade1	
**cGvHD YN**	N	N	N	N	N	N	Y	N	N
**cGVH Grade**							moderate		
**Died**	N	N	N	Y	N	N	N	Y	N
**Relapsed**	N	N	N	Y	N	N	N	Y	N
**# of Samples at Diagnosis**	1	1	1	1	1	1	1	0	1
**Day of Collection Pre-HCT**	143	120	218	100	106	115	203		581

**# of Samples Pre-HCT**	1	1	1	1	1	1	1	1	1
**Days of Collection Pre-HCT (Range)**	22	1	20	21	16	21	27	21	43
**# of Samples Post-HCT**	1	2	2	2	3	12	1	1	1
**Days of Collection Post-HCT (Range)**	20	58-93	79-195	63-105	68-166	32-921	63	203	58
**Median Days**	20	76	137	84	78	249	63	203	58

**UPN**	**37**	**38**	**39**	**40**	**41**	**42**	**43**	**44**	**45**
**Age at BMT**	55	36	43	47	63	51	58	55	65
**Gender**	Female	Female	Male	Female	Male	Female	Female	Female	Female
**Stage**	CR1	CR1	CR1	CR2	CR1	CR1	CR1	CR1	CR1
**DRI (0-4)**	1	1	1	0	1	1	1	1	1
**Donor**	MRD	MRD	URD	URD	MRD	URD	Haplo	MRD	URD
**RIC/MAC**	MAC	MAC	MAC	RIC	RIC	RIC	MAC	MAC	RIC
**GVH Prophylaxis**	ATG-CSA-MTX	PTCy-CSA-MMF	ATG-PTCy-CSA	ATG-PTCy-CSA	ATG-CSA-MTX	ATG-PTCy-CSA	ATG-PTCy-CSA	PTCy-CSA-MMF	ATG-PTCy-CSA
**Graft Source**	PB	PB	PB	BM	PB	PB	PB	PB	PB
**aGvHD YN**	Y	N	N	N	Y	N	N	Y	N
**aGVH Grade**	grade2				grade3			grade2	

**cGvHD YN**	Y	N	N	N	N	N	N	Y	N
**cGVH Grade**	severe							moderate	
**Died**	Y	N	N	Y	N	N	Y	N	N
**Relapsed**	N	N	N	Y	N	N	N	N	N
**# of Samples at Diagnosis**	1	1	1	1	1	1	1	1	1
**Day of Collection Pre-HCT**	110	135	105	612	161	141	125	93	155
**# of Samples Pre-HCT**	1	1	1	1	1	1	0	1	1
**Days of Collection Pre-HCT (Range)**	22	28	14	59	39	16		16	14
**# of Samples Post-HCT**	2	1	1	1	1	4	1	1	1
**Days of Collection Post-HCT (Range)**	29-68	55	64	60	35	104-216	28	41	64
**Median Days**	49	55	64	60	35	139	28	41	64

**UPN**	**46**	**47**	**48**	**49**	**50**	**51**	**52**	**53**	**54**
**Age at BMT**	47	62	68	63	19	69	54	69	63
**Gender**	Female	Male	Male	Male	Female	Female	Female	Female	Female
**Stage**	CR1	CR1	CR2	CR1	CR1	CR1	CR1	CR1	CR1
**DRI (0-4)**	1	3	1	2	1	2	2	1	1
**Donor**	Haplo	URD	MRD	URD	MRD	Haplo	URD	URD	URD

**RIC/MAC**	RIC	RIC	RIC	RIC	RIC	RIC	RIC	RIC	RIC
**GVH Prophylaxis**	ATG-PTCy-CSA	ATG-PTCy-CSA	ATG-PTCy-CSA	ATG-PTCy-CSA	ATG-PTCy-CSA	ATG-PTCy-CSA	ATG-PTCy-CSA	ATG-PTCy-CSA	ATG-PTCy-CSA
**Graft Source**	PB	PB	PB	PB	PB	PB	PB	PB	PB
**aGvHD YN**	Y	Y	Y	N	N	N	N	Y	N
**aGVH Grade**	grade1	grade1	grade2					grade2	
**cGvHD YN**	Y	N	N	N	N	N	N	N	Y
**cGVH Grade**	moderate								moderate
**Died**	N	Y	N	Y	Y	N	N	N	N
**Relapsed**	N	Y	N	Y	Y	N	N	N	N
**# of Samples at Diagnosis**	1	1	1	1	1	1	1	1	1
**Day of Collection Pre-HCT**	265	154	193	90	101	162	175	132	125
**# of Samples Pre-HCT**	1	1	1	1	1	1	1	1	1
**Days of Collection Pre-HCT (Range)**	13	8	11	21	14	23	15	20	28
**# of Samples Post-HCT**	1	1	1	3	3	3	3	2	2
**Days of Collection Post-HCT (Range)**	58	112	63	169-407	61-140	58-205	61-83	31-56	36-64
**Median Days**	58	112	63	392	84	104	76	44	50
**UPN**	**55**	**56**	**57**	**58**	**59**	**60**	**61**	**62**	**63**
**Age at BMT**	68	56	52	63	48	49	65	54	70
**Gender**	Female	Male	Female	Female	Male	Male	Male	Female	Male
**Stage**	CR2	CR1	CR1	CR2	CR1	CR1	CR1	CR1	CR1
**DRI (0-4)**	1	2	1	1	1	1	1	1	2
**Donor**	URD	URD	URD	Haplo	URD	URD	URD	MRD	URD
**RIC/MAC**	RIC	RIC	MAC	RIC	RIC	MAC	RIC	RIC	RIC
**GVH Prophylaxis**	ATG-PTCy-CSA	ATG-PTCy-CSA	ATG-PTCy-CSA	ATG-PTCy-CSA	ATG-CSA-MTX	ATG-PTCy-CSA	ATG-PTCy-CSA	ATG-PTCy-CSA	ATG-PTCy-CSA
**Graft Source**	PB	PB	PB	PB	PB	PB	PB	PB	PB

**aGvHD YN**	N	N	Y	N	Y	N	N	Y	N
**aGVH Grade**			grade2		grade2			grade2	
**cGvHD YN**	N	N	Y	N	N	N	Y	N	N
**cGVH Grade**			moderate				mild		
**Died**	N	N	N	Y	N	N	N	N	Y
**Relapsed**	N	N	N	N	N	Y	N	N	N
**# of Samples at Diagnosis**	1	1	1	1	1	1	1	1	1
**Day of Collection Pre-HCT**	202	156	158	1659	141	150	94	166	163
**# of Samples Pre-HCT**	1	1	1	1	1	1	1	2	1
**Days of Collection Pre-HCT (Range)**	28	10	19	26	17	10	14	22-5	16
**# of Samples Post-HCT**	1	4	2	1	2	1	1	9	2
**Days of Collection Post-HCT (Range)**	107	52-206	25-32	28	70-84	32	175	85-684	69-90
**Median Days**	107	131	29	28	77	32	175	344	80

Abbreviations: aGvHD, acute graft-versus-host disease; ATG, anti-thymocyte globulin; BM, bone marrow; CR1, first complete remission; CR2, second complete remission; CSA, cyclosporine; cGVHD, chronic graft-versus-host disease; Haplo, haploidentical donor (partially matched donor); MAC, myeloablative conditioning; MMF, mycophenolate mofetil; MTX, methotrexate; MUD, matched unrelated donor; PB, peripheral blood; PTCy, post-transplant cyclophosphamide; RIC, reduced-intensity conditioning; and UPN, Unique Patient ID.

Table S2. Clinical Covariates Associated with Overall Survival.

Outcome	Covariate	coef	exp(coef)	se(coef)	*p* value
**Survival**	Gender	−0.714	0.490	1.101	0.517
	Donor Gender	−0.795	0.452	0.731	0.277
	Gender:Donor Gender	0.820	2.271	1.302	0.529
	Age at BMT	−0.074	0.929	0.042	0.076
	**Donor Age**	**−0.210**	**0.811**	**0.098**	**0.032**
	**Age at BMT: Donor Age**	**0.003**	**1.003**	**0.002**	**0.039**
	Recipient CMV	1.482	4.401	1.038	0.154
	Donor CMV	−0.882	0.414	0.531	0.097
	tt(aGvHD)	0.745	2.106	0.725	0.304
	tt(aGvHD Gr0 1)	0.044	1.045	1.240	0.972
	tt(aGvHD Gr2 4)	0.789	2.201	0.747	0.291
	tt(aGvHD Gr3 4)	0.440	1.552	1.139	0.700
	tt(aGvHD GI Gr2 4)	−0.102	0.903	0.726	0.888
	tt(cGvHD)	3.050	21.118	2.310	0.187
	tt(cGvHD Mild)	−0.045	0.956	9066.173	1.000
	tt(cGvHD Mod Sev)	3.472	32.192	2.415	0.151

Cox proportional hazards analysis of recipient and donor characteristics (age, gender, CMV serostatus) and graft-versus-host disease (acute [aGvHD] and chronic [cGvHD]). Significant associations ($p < 0.05$) are highlighted in bold. Interaction terms indicated by a colon (:). Time-dependent variables denoted by tt(x). Abbreviations: BMT, Bone Marrow Transplantation; CMV, Cytomegalovirus; aGvHD, Acute Graft-versus-Host Disease; cGvHD, Chronic Graft-versus-Host Disease; Gr, Grade; GI, Gastrointestinal; Mod Sev, Moderate to Severe.

Table S3. Clinical Covariates Associated with Relapse and Non-Relapse Mortality.

Outcome	Covariate	coef	exp(coef)	se(coef)	pvalue
Relapse	Gender	0.305	1.356	0.744	0.625
	Donor Gender	0.002	1.002	0.856	0.998
	Age at BMT	−0.030	0.971	0.021	0.122
	**Donor Age**	**−0.056**	**0.946**	**0.031**	**0.016**
	Recipient CMV	0.020	1.020	0.817	0.983
	Donor CMV	−0.687	0.503	0.711	0.343
	aGvHD	0.021	1.021	0.641	0.975
	aGvHD Gr0 1	0.320	1.377	1.057	0.784
	aGvHD Gr2 4	−0.085	0.919	0.651	0.895
	aGvHD Gr3 4	−18.286	0.000	7720.512	0
	aGvHD GI Gr2 4	−0.802	0.449	1.064	0.473
	cGvHD	−1.398	0.247	1.054	0.141
	cGvHD Mild	−18.125	0.000	9953.773	0
	cGvHD Mod Sev	−1.048	0.351	1.055	0.264
Non relapse mortality	Donor CMV	−0.753	0.471	0.691	0.258
	Gender	−0.231	0.794	0.693	0.739
	Donor Gender	−0.681	0.506	0.711	0.335
	**Age at BMT**	**0.059**	**1.060**	**0.037**	**0.041**
	Donor Age	0.010	1.010	0.021	0.570
	Recipient CMV	18.525	111,015,608.092	6843.199	0
	aGvHD	−0.329	0.720	0.652	0.580
	aGvHD Gr0 1	−17.129	0.000	5898.086	0
	aGvHD Gr2 4	−0.027	0.973	0.650	0.964
	aGvHD Gr3 4	0.694	2.002	0.813	0.312
	aGvHD GI Gr2 4	0.637	1.890	0.702	0.321
	cGvHD	−1.267	0.282	1.056	0.189
	cGvHD Mild	−18.116	0.000	10,590.916	0
	cGvHD Mod Sev	−0.941	0.390	1.057	0.325

Multivariate Cox regression analysis of recipient and donor characteristics and GvHD with cause-specific outcomes. Significant associations ($P<0.05$) are highlighted in bold. Abbreviations: BMT, Bone Marrow Transplantation; CMV, Cytomegalovirus; aGvHD, Acute Graft-versus-Host Disease; cGvHD, Chronic Graft-versus-Host Disease; Gr, Grade; GI, Gastrointestinal; Mod Sev, Moderate to Severe.

Table S4. Significant Metabolites Affecting Overall Survival.

Outcome	CMPID	Compound	Coef	Exp(coef)	P value	FDR
Diagnosis	C003921	Mannitol	1.142	3.133	0.018	0.960
	C00491	L-cystine	1.328	3.775	0.047	0.960
	C003451	6-Phospho-D-gluconate	−0.448	0.639	0.050	0.960
	C05422	Dehydroascorbate	−1.033	0.356	0.056	0.960
	C00943	3′,5′-Cyclic-IMP	−0.105	0.900	0.058	0.960
	C00072	Ascorbate	0.600	1.821	0.060	0.960
	C06231	Ectoine	−0.720	0.487	0.068	0.960
	C00385	Xanthine	−0.315	0.729	0.074	0.960
	C001581	Citrate	−1.132	0.322	0.076	0.960
Pre-transplant	C00183	L-valine	2.053	7.795	0.009	0.579
	C00392	Mannitol	0.896	2.450	0.013	0.579
	C00082	L-tyrosine	1.763	5.830	0.020	0.579
	C01996	Acetylcholine	1.808	6.097	0.027	0.579
	C03017	acyl-C3-(propionyl-carnitine)	1.184	3.268	0.032	0.579
	C02352	1-4-beta-D-Xylan	2.753	15.686	0.036	0.579
	C00791	Creatinine	1.679	5.361	0.041	0.579
	C00315	Spermidine	−0.683	0.505	0.042	0.579
	C01157	trans-4-Hydroxy-L-proline	−1.585	0.205	0.044	0.579
	C00104	IDP	−1.649	0.192	0.049	0.582
	C00031	D-Glucose	2.282	9.794	0.070	0.679
	C00345	6-Phospho-D-gluconate	−0.361	0.697	0.072	0.679
	C00245	Taurine	−0.930	0.395	0.074	0.679
	C00148	L-proline	1.433	4.190	0.084	0.717
	C00123	L-leucine	1.206	3.340	0.096	0.722
	C00327	L-Citrulline	1.630	5.105	0.097	0.722
Post-transplant	**C01602**	**Ornithine**	**−2.757**	**0.063**	**0.001**	**0.089**
	**C06336**	**3-Sulfocatechol**	**−0.528**	**0.590**	**0.004**	**0.226**
	**C00954**	**Indole-3-acetate**	**−1.024**	**0.359**	**0.006**	**0.240**
	C001481	L-proline	−2.673	0.069	0.010	0.297
	C00499	Allantoate	−2.336	0.097	0.015	0.332
	C003271	L-Citrulline	−1.596	0.203	0.017	0.332
	C00009	Phosphate	2.517	12.397	0.020	0.332
	C20826	acyl-C5-(isovalerylcarnitine)	−1.050	0.350	0.024	0.332
	C03451	(R)-S-Lactoylglutathione	−0.637	0.529	0.025	0.332
	C002451	Taurine	−1.744	0.175	0.028	0.339

Cox regression analysis of metabolites from diagnosis, pre-transplant, and post-transplant samples associated with overall survival. Significant associations ($P<0.05$) are highlighted in bold.; adjusted False Discovery Rates (FDR) included. Abbreviations: CMPID, Compound Identification; IMP, Inosine Monophosphate; IDP, Inosine Diphosphate;

Table S5. Pre-transplant Metabolites Predictive of Cause-Specific Mortality.

Outcome	cmpdID	Compound	coef	exp(coef)	pvalue	FDR
Relapse	**C00123**	**L-leucine**	**2.375**	**10.755**	**0.001**	**0.111**
	**C00079**	**L-phenylalanine**	**3.280**	**26.585**	**0.001**	**0.111**
	C00183	L-valine	2.283	9.809	0.032	0.328
	**C01157**	**trans-4-Hydroxy-L-proline**	**-2.290**	**0.101**	**0.003**	**0.171**
	C00104	IDP	3.061	21.352	0.085	0.521
	**C00331**	**Indolepyruvate**	**2.038**	**7.676**	**0.005**	**0.171**
	**C00791**	**Creatinine**	**2.630**	**13.874**	**0.005**	**0.171**
	C00647	Pyridoxamine-5′-phosphate	1.011	2.748	0.100	0.528
	**C00148**	**L-proline**	**1.964**	**7.128**	**0.012**	**0.209**
	**C00943**	**3′,5′-Cyclic-IMP**	**3.087**	**21.919**	**0.013**	**0.209**
	**C00392**	**Mannitol**	**1.100**	**3.005**	**0.013**	**0.209**
	**C00082**	**L-tyrosine**	**1.927**	**6.870**	**0.013**	**0.209**
	C00078	L-tryptophan	1.697	5.455	0.020	0.305
	C01185	Nicotinate-ribonucleotide	1.287	3.622	0.029	0.328
	C00637	Indole-3-acetaldehyde	1.628	5.095	0.029	0.328
	C00366	Urate	2.928	18.682	0.029	0.328
	C01996	Acetylcholine	2.172	8.774	0.030	0.328
	C03451	(R)-S-Lactoylglutathione	0.586	1.796	0.040	0.386
	C01419	Cys-Gly	1.157	3.181	0.041	0.386
	HMDB00651	acyl-C10(O-Decanoyl-L-carnitine)	0.898	2.454	0.046	0.422
	C00245	Taurine	-1.292	0.275	0.051	0.435
	C00097	L-cysteine	1.105	3.020	0.077	0.508
	C02352	1-4-beta-D-Xylan	3.501	33.135	0.078	0.508
	C06428	Eicosapentaenoic-acid	0.565	1.759	0.079	0.508
	C00041	L-alanine	1.270	3.560	0.079	0.508
Nonrelapse	C00123	L-leucine	1.136	3.114	0.060	0.475
	C00183	**L-valine**	**3.197**	**24.454**	**0.003**	**0.171**
	C00104	**IDP**	**-2.396**	**0.091**	**0.004**	**0.171**
	C00647	**Pyridoxamine-5′-phosphate**	**-1.161**	**0.313**	**0.006**	**0.171**
	C00327	**L-Citrulline**	**3.019**	**20.478**	**0.008**	**0.204**
	**C01879**	**5-Oxoproline**	**2.465**	**11.766**	**0.010**	**0.209**
	**C00064**	**L-glutamine**	**2.163**	**8.701**	**0.013**	**0.209**
	C00082	L-tyrosine	1.883	6.572	0.092	0.521
	C00009	Phosphate	1.328	0.265	0.029	0.328
	C00315	Spermidine	-0.892	0.410	0.030	0.328
	C06231	Ectoine	-1.052	0.349	0.049	0.430
	C00026	2-Oxoglutarate	-1.350	0.259	0.054	0.443
	C00119	5-Phospho-alpha-D-ribose-1-diphosphate	-1.640	0.194	0.066	0.490
	C00906	5-6-Dihydrothymine	0.912	2.490	0.066	0.490
	C01684	D-Rhamnose	-0.684	0.505	0.069	0.497
	C03017	acyl-C3(propionyl-carnitine)	1.074	2.926	0.090	0.521
	C00262	Hypoxanthine	0.934	2.546	0.090	0.521
	C00385	Xanthine	-1.616	0.199	0.091	0.521
	C00345	6-Phospho-D-gluconate	-0.429	0.651	0.100	0.528

List of pre-transplant metabolites significantly associated (P<0.05) with relapse-related and non-relapse mortality. Adjusted False Discovery Rates (FDR) provided. Significant associations (P<0.05) are highlighted in bold. Abbreviations: CMPID, Compound Identification; IMP, Inosine Monophosphate; IDP, Inosine Diphosphate;

Table S6. Post-transplant Metabolites Predictive of Cause-Specific Mortality.

Outcome	cmpdID	Compound	coef	exp(coef)	*p*-value	FDR
Relapse	C05422	Dehydroascorbate	−3.428	0.032	0.002	0.354
	C06231	Ectoine	0.614	1.848	0.011	0.354
	C00036	Oxaloacetate	4.314	74.754	0.017	0.354
	C00158	Citrate	−2.263	0.104	0.023	0.354
	C00122	Fumarate	−1.603	0.201	0.028	0.354
	C00864	Pantothenate	−0.964	0.381	0.029	0.354
	C00647	Pyridoxamine-5′-phosphate	0.988	2.685	0.033	0.373
	C00149	Malate	−2.477	0.084	0.038	0.402
	C00065	L-serine	−1.721	0.179	0.044	0.402
	C00245	Taurine	−2.101	0.122	0.057	0.488
	HMDB00756	acyl-C6(hexanoyl-l-carnitine)	−0.536	0.585	0.061	0.504
	CID57357170	acyl-C10:1(O-Decenoyl-L-carnitine)	−0.711	0.491	0.073	0.543
	C00042	Succinate	−2.109	0.121	0.081	0.572
	C02700	N−formyl−kynurenine	1.802	6.063	0.094	0.596
Nonrelapse	C01602	Ornithine	−2.791	0.061	0.004	0.354
	C06336	3−Sulfocatechol	−0.616	0.540	0.006	0.354
	C01684	D−Rhamnose	−1.200	0.301	0.012	0.354
	C03451	(R)−S−Lactoylglutathione	−0.883	0.414	0.014	0.354
	C00328	kynurenine	2.694	14.792	0.014	0.354
	C00637	Indole−3−acetaldehyde	−2.144	0.117	0.019	0.354
	C00242	Guanine	1.238	3.448	0.019	0.354
	C00078	L−tryptophan	−2.732	0.065	0.019	0.354
	C00219	Eicosatetraenoic−acid	1.591	4.907	0.023	0.354
	C01571	Decanoic−acid(caprate)	−1.769	0.170	0.026	0.354
	C00463	Indole	−1.673	0.188	0.027	0.354
	C00499	Allantoate	−2.508	0.081	0.028	0.354
	C00101	Tetrahydrofolate	−0.972	0.378	0.030	0.354
	C00315	Spermidine	−1.038	0.354	0.030	0.354
	C01595	Linoleate	1.101	3.008	0.039	0.402
	C20826	acyl−C5(isovalerylcarnitine)	−1.117	0.327	0.042	0.402
	C00009	Phosphate	3.189	24.263	0.044	0.402
	C00385	Xanthine	−2.449	0.086	0.054	0.477
	C00712	Octadecenoic−acid	1.486	4.420	0.066	0.522
	C00345	6−Phospho−D−gluconate	−0.702	0.496	0.068	0.523
	C03453	g−Oxalo−crotonate	1.621	5.057	0.082	0.572
	C05695	gamma−Glutamyl−Se−methy lselenocysteine	−2.104	0.122	0.085	0.574
	C00148	L−proline	−4.160	0.016	0.087	0.574
	C03722	quinolinic−acid	0.969	2.636	0.095	0.596

List of post−transplant metabolites significantly associated (P<0.05) with relapse−related and non−relapse mortality. Adjusted False Discovery Rates (FDR) provided. Abbreviations: CMPID, Compound Identification.

Table S7. Diagnostic Metabolites Predictive of Cause−Specific Mortality.

Outcome	cmpdID	Compound	coef	exp(coef)	*p*-value	FDR
Relapse	C02989	L−Methionine−S−oxide	−1.490	0.225	0.002	0.365
	C01595	Linoleate	−1.201	0.301	0.007	0.365
	C00082	L−tyrosine	−3.027	0.048	0.011	0.365
	C00158	Citrate	−1.856	0.156	0.013	0.365
	C05422	Dehydroascorbate	−2.240	0.106	0.014	0.365
	C00245	Taurine	3.600	36.613	0.033	0.602
	C00954	Indole−3−acetate	−0.909	0.403	0.081	0.713
	C01530	Octadecanoic−acid	−1.879	0.153	0.035	0.602
	C00712	Octadecenoic−acid	−0.976	0.377	0.051	0.681
	C00791	Creatinine	−1.360	0.257	0.051	0.681
	C05283	(5−L−Glutamyl)−L−glutamine	−0.795	0.452	0.059	0.698
	C00249	Hexadecanoic−acid	−1.212	0.298	0.073	0.713
	C00300	Creatine	0.516	1.675	0.084	0.713
Nonrelapse	C02989	L−Methionine−S−oxide	1.189	3.283	0.083	0.713
	C00491	L−cystine	1.893	6.637	0.005	0.365
	C00345	6−Phospho−D−gluconate	−0.821	0.440	0.007	0.365
	C00072	Ascorbate	0.851	2.341	0.008	0.365
	HMDB00651	acyl−C10(O−Decanoyl−L−carnitine)	−0.834	0.434	0.011	0.365
	HMDB00791	acyl−C8(L−octanoylcarnitine)	−0.783	0.457	0.019	0.454
	C00954	Indole−3−acetate	1.306	3.693	0.034	0.602
	C00392	Mannitol	1.107	3.024	0.035	0.602
	C00119	5−Phospho−alpha−D−ribose−1−diphosphate	2.114	8.279	0.039	0.619
	HMDB02250	acyl−C12(O−dodecanoyl−carnitine)	−0.661	0.516	0.042	0.632
	HMDB00756	acyl−C6(hexanoyl−l−carnitine)	−0.606	0.546	0.059	0.698
	HMDB05066	acyl−C14(O−tetradecanoyl−L−carnitine)	−0.598	0.550	0.063	0.698
	C00250	Pyridoxal	0.539	1.715	0.065	0.698
	C00135	L−histidine	2.470	11.824	0.075	0.713
	C00042	Succinate	−0.857	0.425	0.084	0.713

List of metabolites measured at diagnosis significantly associated (P<0.05) with relapse−related and non−relapse mortality. Adjusted False Discovery Rates (FDR) provided.
Abbreviations: CMPID, Compound Identification;

Table S8. Top Compounds Associated with Relapse.

cmpdID	Compound	coef	T value	*p*-value	FDR
C05422	**Dehydroascorbate**	**−0.31**	**−3.89**	**1.30e−04**	**0.02**
C00158	**Citrate**	**−0.23**	**−2.97**	**3.26e−03**	**0.19**
C01904	D−Arabitol	−0.21	−2.57	1.08e−02	0.40
C00864	Pantothenate	−0.38	−2.45	1.51e−02	0.40
C00149	Malate	−0.24	−2.40	1.70e−02	0.40
C00262	Hypoxanthine	−0.30	−2.21	2.81e−02	0.51
C00122	Fumarate	−0.27	−2.10	3.71e−02	0.51
CID57357170	acyl−C10:1(O−Decen oyl−L−carnitine)	−0.35	−2.08	3.81e−02	0.51
C01595	Linoleate	−0.31	−2.08	3.87e−02	0.51
C00062	L−arginine	0.19	2.03	4.30e−02	0.51

Results from linear mixed−effects model analysis comparing metabolite levels between relapse and non−relapse patients across diagnosis, pre−transplant, and post−transplant time points. Significant associations ($P<0.05$) are highlighted in bold, along with corresponding regression coefficients, t−values, unadjusted P−values, and False Discovery Rates (FDR). Abbreviations: CMPID, Compound Identification.
